# Single-Frequency Precise Point Positioning Using Regional Dual-Frequency Observations

**DOI:** 10.3390/s21082856

**Published:** 2021-04-18

**Authors:** Junping Zou, Ahao Wang, Jiexian Wang

**Affiliations:** 1 College of Surveying and Geo-Informatics, Tongji University, Shanghai 200092, China; 1410902@tongji.edu.cn (J.Z.); wangjiexian@tongji.edu.cn (J.W.); 2 College of Geoscience and Surveying Engineering, China University of Mining and Technology-Beijing, Beijing 100083, China

**Keywords:** precise point positioning, single-frequency positioning, global positioning system, ionosphere delay

## Abstract

High-precision and low-cost single-frequency precise point positioning (SF-PPP) has been attracting more and more attention in numerous global navigation satellite system (GNSS) applications. To provide the precise ionosphere delay and improve the positioning accuracy of the SF-PPP, the dual-frequency receiver, which receives dual-frequency observations, is used. Based on the serviced precise ionosphere delay, which is generated from the dual-frequency observations, the high-precision SF-PPP is realized. To further improve the accuracy of the SF-PPP and shorten its convergence time, the double-differenced (DD) ambiguity resolutions, which are generated from the DD algorithm, are introduced. This method avoids the estimation of fractional cycle bias (FCB) for the SF-PPP ambiguity. Here, we collected data from six stations of Shanghai China which was processed, and the corresponding results were analyzed. The results of the dual-frequency observations enhanced SF-PPP realize centimeter-level positioning. The difference between the results of two stations estimated with dual-frequency observations enhanced SF-PPP were compared with the relative positioning results computed with the DD algorithm. Experimental results showed that the relative positioning accuracy of the DD algorithm is slightly better than that of the dual-frequency observations enhanced SF-PPP. This could be explained by the effect of the float ambiguity resolutions on the positioning accuracy. The data was processed with the proposed method for the introduction of the DD ambiguity into SF-PPP and the results indicated that this method could improve the positioning accuracy and shorten the convergence time of the SF-PPP. The results could further improve the deformation monitoring ability of SF-PPP.

## 1. Introduction

It is true that the single-frequency (SF) global navigation satellite system (GNSS) receiver is widely used, especially in the positioning, timing, and navigation (PNT) service. With the rapid development of smart devices such as smartphones, shared bicycles, and personal digital assistants, more and more low-cost GNSS chipsets receiving SF signals are embedded in these advanced devices. Some researchers have carried out testing, and quality assessments of multi-GNSS raw measurements from different types of smart devices, and mass-market GNSS chipsets can achieve submeter- or decimeter-level positioning accuracy [1,2,3,4,5]. High-precision and low-cost single-frequency precise point positioning (SF-PPP) has attracted increasing attention in the booming GNSS markets. However, space-dependent errors, such as ionosphere delay and troposphere delay, affect the positioning performance. These errors are generally eliminated using the differenced algorithm or correction information. In regards to the relative positioning of the SF receiver, two receivers at least are needed, and the relative positioning results are easily affected by the displacement of the localized infrastructure. Therefore, the ability of displacement detection by relative positioning is limited [6]. Different from the relative positioning, the service of the correction information for the ionosphere delay, which is estimated using the GNSS geometry-free combination, is needed for single-frequency and single-receiver-based absolute positioning. This correction information is generally realized by modeling the ionosphere delay estimated with code geometry-free combination formed with P1 and P2 observations from local or regional network stations [7,8,9]. It is obvious that the estimated ionosphere delay is easily affected by the noise of code observations and the density of used observations. Besides, the ionosphere delay can be removed with the GRAPHIC (Group and Phase Ionosphere Calibration) combination, but it makes the convergence time longer since the design matrix of the combination is similar to that of the phase observation [10]. Wang et al. [11] compared and evaluated the performance of the three widely used SF-PPP models; the kinematic test results showed that the positioning accuracy and convergence of the ionosphere-weighted SF-PPP model was better than that of the traditional ionosphere-corrected and GRAPHIC models. The ionosphere delay error could be extracted with undifferenced and uncombined PPP [12]. In this method, raw phase and code observations were used to estimate the ionosphere model parameters, but the receiver differential code bias (DCB) was just computed, and the satellite DCB was corrected using International GNSS Service (IGS) [13] products. Beyond that, the tedious process for ionosphere modeling complicates its application. The precise ionosphere delay was estimated and used in the single-frequency receiver positioning based on the local network observations in Deng et al. [14] and Zou et al. [15]. In SF-PPP, the ambiguity still needs to be processed. The existence of the satellite fractional cycle bias (FCB) makes single-differenced (SD) ambiguity between two satellites have no integer property [16], while the integer ambiguity resolution is very important to shorten the convergence time and improve the positioning accuracy of SF-PPP. The estimation of FCB and ambiguity fixing of SF-PPP is similar to that of PPP. In processing, the FCB should be estimated and serviced. The PPP-enabled ambiguity resolution has been studied and implemented [6,16,17,18,19,20,21,22,23,24,25,26,27,28], and these studies focus on the recovering of the integer characteristic. In Li et al. [6] and Odijk et al. [29], the double-differenced ambiguity was introduced in PPP ambiguity processing to improve the positioning accuracy of PPP and promote the detecting ability of the PPP in displacement monitoring. This method avoids the estimation of the FCB, and the PPP ambiguity fixing was realized based on the introduced SD ambiguity from reference stations and the double-differenced ambiguity fixing.

At present, with SF-PPP technology, it is difficult to meet the requirements of centimeter-level positioning since the fast fixed ambiguity cannot be realized, and the accuracy of the external ionosphere models is limited. Therefore, it is very important and beneficial to introduce the double-differenced ambiguity into SF-PPP to realize high-precision positioning applications. It will promote the application of the single-frequency receiver in displacement monitoring. The accuracy of SF-PPP is easily affected by serviced ionosphere delay, so it is important to provide precise service for ionosphere correction. The precise ionosphere delay can be extracted by using dual-frequency observations. Thus, a method for the dual-frequency observations enhanced SF-PPP is presented. The method includes: (1) Estimation of the precise ionosphere delay for single-frequency receiver; (2) Processing of SF-PPP; (3) double-differenced ambiguities estimated based on the double-differenced single-frequency observations; (4) undifferenced and satellite-differenced ambiguities generated based on SF-PPP computation; (5) double-differenced and satellite-differenced ambiguities introduced in SF-PPP. After the abovementioned five steps are taken, SF-PPP is realized based on the precise service of the ionosphere delay. The high-precision coordinate results of SF-PPP were obtained by introducing the double-differenced ambiguity. In the following, the “Materials and Methods” Section introduces the method for estimation of the precise ionosphere delay and the introduction of the double-differenced integer ambiguity resolution into SF-PPP. The “Data and Experiment” and “Discussion” Sections indicate the data analysis and discuss the results. Finally, the “Conclusions” Section summarizes the main findings.

## 2. Materials and Methods

Here, traditional SF-PPP is discussed, and the methods for the estimation and interpolation of the precise ionosphere delay are presented. With the interpolated precise ionosphere delay, the SF-PPP of the user could be implemented. Then the double-differenced (DD) ambiguity was introduced into SF-PPP to fix the ambiguity.

### 2.1. SF-PPP

Assuming that the residual errors of satellite orbit and clock are neglected, for a single-receiver, if the corrections for the Earth rotation, Earth tides, relativistic effects, phase center variation (PCV), and DCB are implemented, the observed-corrected L1 and P1 observations are written as in Equation (1):

(1) $$\begin{array}{l} P_{1}=\rho +b_{1}^{r}-b_{1}^{s}+\delta_{r}+I_{r}^{s}+T_{r}^{s}+\omega_{1}\\ L_{1}=\rho +N_{1}\lambda_{1}+(FCB_{1}^{r}-FCB_{1}^{s})\lambda_{1}+\delta_{r}-I_{r}^{s}+T_{r}^{s}+\varepsilon_{1} \end{array}$$

where *ρ* is the geometric range between the receiver and satellite, $T_{r}^{s}$ is the slant tropospheric delay, $I_{r}^{s}$ is the slant ionospheric delay of the *L*_1_ observation, $\delta_{r}$ is the receiver clock errors in meter, $b_{1}^{s}$ is the satellite hardware delay biases of the P1 observation, $b_{1}^{r}$ is the receiver hardware delay biases of P1 observation, $\varepsilon_{1}$ is the noise from the *L*_1_ observation, $\omega_{1}$ is the noise from the P1 observation; $FCB_{1}^{r}$ is the receiver FCB and $FCB_{1}^{s}$ is the satellite FCB of the *L*_1_ observation; $N_{1}$ is the integer ambiguities of *L*_1_ and its wavelength is $\lambda_{1}$. In SF-PPP processing, the ionosphere delay is generally corrected with the established model using data from ground reference stations, in which the precise satellite clock and orbit products are used [30,31,32,33]. The commonly used models are the Klobuchar Model and Global Ionospheric Model (GIM) provided by IGS [34,35]. However, the accuracy of the serviced ionosphere delay is too low to meet the need for precise positioning. Thus, the precise ionosphere delay is estimated using the regional dual-frequency observations [14,15].

### 2.2. Estimation and Interpolation of the Precise Ionosphere Delay

The precise ionosphere delay is estimated with dual-frequency observation [14,15]. For a dual-frequency user, the geometry-free phase and code observation can be calculated using Equation (2)

(2) $$\begin{array}{c} L_{1}-L_{2}=N_{1}\lambda_{1}-N_{2}\lambda_{2}+(FCB_{1}^{r}-FCB_{1}^{s})\lambda_{1}-(FCB_{2}^{r}-FCB_{2}^{s})\lambda_{2}-\frac{f_{2}^{2}-f_{1}^{2}}{f_{2}^{2}}I_{r}^{s}+\varepsilon_{1}-\varepsilon_{2}\\ P_{1}-P_{2}=b_{1}^{r}-b_{2}^{r}-(b_{1}^{s}-b_{2}^{s})+\frac{f_{2}^{2}-f_{1}^{2}}{f_{2}^{2}}I_{r}^{s}+\omega_{1}-\omega_{2}\\ =DCB{\left(P_{1}-P_{2}\right)}^{r}-DCB{\left(P_{1}-P_{2}\right)}^{s}+\frac{f_{2}^{2}-f_{1}^{2}}{f_{2}^{2}}I_{r}^{s}+\omega_{1}-\omega_{2} \end{array}$$

where $N_{2}$ is the integer ambiguity of the *L_2_* observation and its wavelength is $\lambda_{2}$; $FCB_{2}^{r}$ is the receiver FCB and $FCB_{2}^{s}$ is the satellite FCB of the *L_2_* observation; $b_{2}^{s}$ is the satellite hardware delay biases of the *P*_2_ observation; $b_{2}^{r}$ is the receiver hardware delay biases of the *P*_2_ observation; *f*_1_ and *f*_2_ are the frequencies of *L*_1_ and *L*_2_ observations; $\varepsilon_{2}$ is the noise of *L_2_* observation; $\omega_{2}$ is the noise of *P*_2_ observation; $DCB{\left(P_{1}-P_{2}\right)}^{s}$ is the satellite DCB and $DCB{\left(P_{1}-P_{2}\right)}^{r}$ is the receiver DCB [36]. Equation (3) shows that when there is no cycle slip between two adjacent epochs, the precise time-varying ionosphere delay can be computed with geometry-free phase observation and the epoch-differenced algorithm between epoch *n* and *n−*1:

(3) $${\left(∆I_{r}^{s}\right)}_{n}=\frac{f_{2}^{2}}{f_{1}^{2}-f_{2}^{2}}∆{\left(L_{1}-L_{2}\right)}_{n}=\frac{f_{2}^{2}}{f_{1}^{2}-f_{2}^{2}}({\left(L_{1}-L_{2}\right)}_{n}-\left(L_{1}-L_{2}{)}_{n-1}\right)$$

The cycle slip is detected using the geometry-free phase and the Hatch–Melbourne–Wübbena observation combinations [37,38,39]. When the reference epoch *k* is selected, the reference epoch based ionosphere delay at epoch *k* + *m* can be computed with Equation (4):

(4) $${\left(I_{r}^{s}\right)}_{k+m}=I_{r,k}^{s}+\sum_{i=k+1}^{k+m}{\left(∆I_{r}^{s}\right)}_{i}$$

where $I_{r,k}^{s}$ is the ionospheric delay at the reference epoch of *k*. The reference epoch can be selected when the satellite is tracked for the first time or the cycle slips happen. Therefore, $I_{r,k}^{s}$ is computed for each successive period of epochs without cycle slip. When the estimated ionosphere delay with Equation (4) is introduced in the geometry-free code observation, the geometry-free code observation can be re-written as Equation (5):

(5) $$\begin{array}{c} {\left(P_{1}-P_{2}\right)}_{k+m}=DCB{\left(P_{1}-P_{2}\right)}^{r}-DCB{\left(P_{1}-P_{2}\right)}^{s}\\ +\frac{f_{2}^{2}-f_{1}^{2}}{f_{2}^{2}}\left(I_{r,k}^{s}+\overset{k+m}{\underset{i=k+1}{\sum }}{\left(∆I_{r}^{s}\right)}_{i}\right)+\omega_{1}-\omega_{2} \end{array}$$

Equation (5) shows that the ionosphere delay at the reference epoch of *k* can be computed when the epoch-differenced ionosphere delays estimated with geometry-free phase observation are obtained:

(6) $$\begin{array}{c} \left(I_{r,k}^{s}-\frac{f_{2}^{2}}{f_{1}^{2}-f_{2}^{2}}\left(DCB{\left(P_{1}-P_{2}\right)}^{r}-DCB{\left(P_{1}-P_{2}\right)}^{s}\right)\right)\\ =-\overset{k+m}{\underset{i=k+1}{\sum }}{\left(∆I_{r}^{s}\right)}_{i}-\frac{f_{2}^{2}}{f_{1}^{2}-f_{2}^{2}}{\left(P_{1}-P_{2}\right)}_{k+m}+\omega_{1}-\omega_{2} \end{array}$$

The accuracy of the epoch-differenced ionosphere delays estimated with geometry-free phase observation is relatively high. The effect of the code noise on the computed ionosphere delay at the reference epoch is deduced by averaging the observations of the many epochs. Therefore, Equation (6) can be written as Equation (7):

(7) $$\begin{array}{c} \left(I_{r,k}^{s}-\frac{f_{2}^{2}}{f_{1}^{2}-f_{2}^{2}}\left(DCB{\left(P_{1}-P_{2}\right)}^{r}-DCB{\left(P_{1}-P_{2}\right)}^{s}\right)\right)\\ =-\frac{1}{m+1}(\overset{k+m}{\underset{i=k+1}{\sum }}(\left(m+k+1-i\right)\left(∆I_{r}^{s}{)}_{i}\right)+\frac{f_{2}^{2}}{f_{1}^{2}-f_{2}^{2}}\overset{k+m}{\underset{i=k+0}{\sum }}{\left(P_{1}-P_{2}\right)}_{i})+\omega_{1}-\omega_{2} \end{array}$$

Equation (7) shows the ionosphere delay at the reference epoch of *k* absorbs the receiver and satellite DCBs. The satellite DCBs used here are products released by CODE at intervals of days. As for the receiver DCB, it is absorbed by the receiver clock in the positioning processing so that it will not affect the positioning result [36]. In near-real-time mode, the satellite DCBs can be predicted with the values of the day before since DCB values change little between two adjacent days. The ionosphere delay is the space-dependent error. Thus, the precise ionosphere delay at the user can be interpolated using the inverse distance weighted method after the ionosphere delay at the reference epoch of *k* and the accumulated epoch-differenced ionosphere delay computed with geometry-free phase observation are obtained. The inverse distance weighted method [6] is calculated by the following:

(8) $$\begin{array}{c} {\left(I_{r,k}^{s}\right)}_{u}=\overset{g}{\underset{j=1}{\sum }}q_{j}{\left(I_{r,k}^{s}\right)}_{j}/\overset{g}{\underset{j=1}{\sum }}\left(q_{j}\right) \end{array}$$

(9) $$\begin{array}{c} q_{j}=\frac{1}{l_{j}} \end{array}$$

where *g* is the total number of reference stations; ${\left(I_{r,k}^{s}\right)}_{u}$ is the interpolated precise ionosphere delay at single-frequency user of *u*; $q_{j}$ is the weight; $l_{j}$ is the distance between the dual- and single-frequency user stations.

### 2.3. Introduction of the DD Ambiguity into SF-PPP

**Figure 1:**
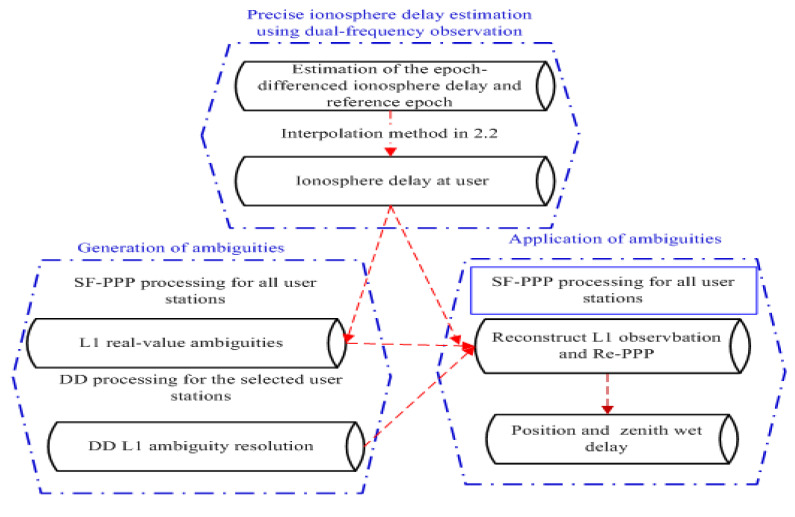
Flowchart of the estimation and interpolation of the precise ionosphere delay and the introduction of the DD ambiguity into SF-PPP.

When the precise ionosphere delay at the user is obtained, the single-frequency user can carry out the PPP computation. Equation (1) shows that the ambiguity of the L1 observation absorbs the satellite FCB so that it cannot be fixed to an integer value, and the ambiguity fixing method is not suitable for implementation [6]. The ambiguity fixing is beneficial to improve the positioning accuracy and shorten convergence time. To improve the positioning accuracy of the SF-PPP, its ambiguity needs to be fixed by introducing the DD ambiguity into it. The satellite-differenced (SD) L1 observation of the satellites *a* and *b* at user is written as in [6]:

(10) $$\begin{array}{c} L_{1,r}^{a}-L_{1,r}^{b}=∆\rho +\left(N_{1,r}^{a}-N_{1,r}^{b}\right)\lambda_{1}-\left(FCB_{1}^{a}-FCB_{1}^{b}\right)\lambda_{1}\\ -\begin{array}{c} (I_{r}^{a}-I_{r}^{b})+\left(T_{r}^{a}-T_{r}^{b}\right) \end{array}-\left(\delta_{s}^{a}-\delta_{s}^{b}\right)+\varepsilon_{1,a}-\varepsilon_{1,b}\\ =L_{1,r}^{a,b}=∆\rho +N_{1,r}^{a,b}\lambda_{1}-FCB_{1}^{a,b}\lambda_{1}-∆I_{r}^{a,b}+∆T_{r}^{a,b}-\left(\delta_{s}^{a}-\delta_{s}^{b}\right)+\varepsilon_{a,b} \end{array}$$

where $\delta_{s}^{a}$ and $\delta_{s}^{b}$ are satellite clock errors in meters. Equation (10) shows that the SD algorithm cancels the receiver FCB. When a high-quality SD real-value *L*_1_ ambiguity, which has the best performance in PPP and DD positioning, at another receiver *u* is introduced, the SD satellite FCB is removed, and Equation (10) can be re-written as:

(11) $$\begin{array}{c} L_{1,r}^{a,b}=∆\rho +N_{1,r}^{a,b}\lambda_{1}-FCB_{1}^{a,b}\lambda_{1}-I_{r}^{a,b}+T_{r}^{a,b}-\left(\delta_{s}^{a}-\delta_{s}^{b}\right)+\varepsilon_{a,b}\\ -\left(N_{1,u}^{a,b}\lambda_{1}-FCB_{1}^{a,b}\lambda_{1}\right)+\left(N_{1,u}^{a,b}\lambda_{1}-FCB_{1}^{a,b}\lambda_{1}\right)\\ =∆\rho +N_{1,r}^{a,b}\lambda_{1}-N_{1,u}^{a,b}\lambda_{1}+\left(N_{1,u}^{a,b}\lambda_{1}-FCB_{1}^{a,b}\lambda_{1}\right)-∆I_{r}^{a,b}+∆T_{r}^{a,b}-\left(\delta_{s}^{a}-\delta_{s}^{b}\right)+\varepsilon_{a,b}\\ =∆\rho +(N_{1,r}^{a,b}-N_{1,u}^{a,b})\lambda_{1}+\left(N_{1,u}^{a,b}\lambda_{1}-FCB_{1}^{a,b}\lambda_{1}\right)-∆I_{r}^{a,b}+∆T_{r}^{a,b}-\left(\delta_{s}^{a}-\delta_{s}^{b}\right)+\varepsilon_{a,b}\\ =∆\rho +∆\nabla N_{r,u}^{a,b}\lambda_{1}+\left(N_{1,u}^{a,b}\lambda_{1}-FCB_{1}^{a,b}\lambda_{1}\right)-∆I_{r}^{a,b}+∆T_{r}^{a,b}-\left(\delta_{s}^{a}-\delta_{s}^{b}\right)+\varepsilon_{a,b} \end{array}$$

where the term of $N_{1,u}^{a,b}\lambda_{1}-FCB_{1}^{a,b}\lambda_{1}$ is the SD real-value *L*_1_ ambiguity at user *u*, $∆\nabla N_{r,u}^{a,b}$ is the DD integer ambiguity formed with the two users of *r* and *u*. For the determination of the best performance and the selection of the high-quality SD real-value *L*_1_ ambiguity, the ratio values in the DD processing can be used [6]. Equation (11) shows the ambiguity at user has the integer characteristic and can be fixed. The generated ambiguity in Equation (11) is obtained using the DD observation formed by the users *u* and *r*. Figure 1 indicates the flowchart for the estimation and interpolation of the precise ionosphere delay and the introduction of the DD ambiguity into SF-PPP. The SF-PPP processing for all users is carried out, and the corresponding SD float ambiguity is obtained at first. Then, the DD ambiguity is generated as shown in Equation (11), and the ambiguity processing for the SF-PPP is converted into DD ambiguity processing, which is formed with the users of *a* and *b*. When this DD ambiguity is fixed and then introduced into the SF-PPP processing for the user *a* or *b*, the fixed SF-PPP ambiguity is realized.

## 3. Data and Experiment

**Figure 2:**
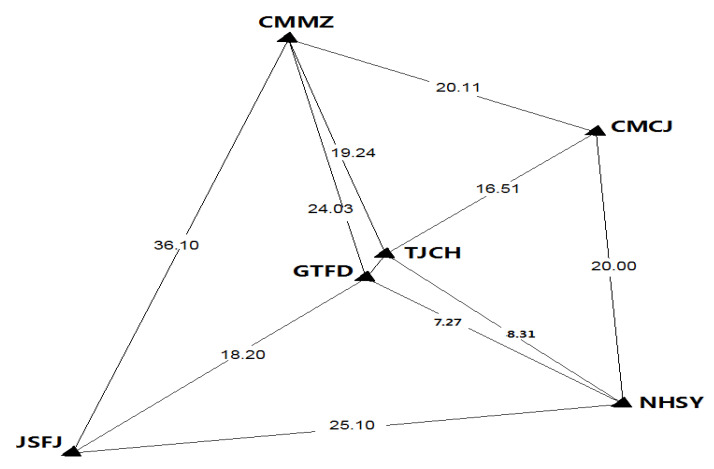
Stations distribution and inter-station distance (km).

To verify the proposed method, 24 h GPS observations with the sampling interval of 30 s from six stations of Shanghai (CMCJ, CMMZ, GTFD, JSFJ, NHSY, TJCH), China, were used. The elevation cut-off angle is set to 9 degrees. Note that the stations TJCH and GTFD received the dual-frequency observations. The distribution of these selected stations is shown in Figure 2.

According to the processing strategy, the dual-frequency observation stations TJCH and GTFD were used to compute and interpolate the precise ionosphere delay for the users of CMCJ, CMMZ, JSFJ, and NHSY. In the precise ionosphere delay computing, the cycle slip is detected using the geometry-free phase and the Hatch–Melbourne–Wübbena observation combinations [37,38,39]. After the single-frequency user obtained the precise ionosphere delay, the SF-PPP is carried out. In SF-PPP processing, the final IGS products of the satellite clock and orbit and the elevation-dependent function are used [40]:

(12) $$\begin{array}{c} w\left(\theta_{j}\right)=\left\{\begin{array}{cc} 1/\sigma^{2} & 30^{0}\leq \theta_{j}\leq 90^{0}\\ 2\sin\left(\theta_{j}\right)/\sigma^{2} & 10^{0}\leq \theta_{j}<30^{0} \end{array}\right\} \end{array}$$

where *θ_j_* is the satellite elevation at epoch *j*; *σ* are the standard deviations in the zenith of phase and code observations. In this processing, the L1 ambiguity, which absorbs the satellite FCB and does not have the integer characteristic, is resolved. Thus, this L1 ambiguity cannot be fixed to an integer value. To improve the accuracy of the SF-PPP, the ambiguity fixing of the SF-PPP was realized based on the introduction of the DD ambiguity into SF-PPP. The introduction of the DD ambiguity into SF-PPP is shown in Figure 1. The corrections for the Earth rotation, Earth tides, relativistic effects, phase center variation (PCV) and DCB were implemented in [41]. The troposphere delay is corrected using the Saastamoinen model, and the residual wet part is estimated by setting up a Piece Wise Constant (PWC) at an interval of 1 h. The adopted models and strategies for SF-PPP in processing software are shown in Table 1 [41]. The 24 h observations at the users of the CMCJ, CMMZ, JSFJ, and NHSY were split into eight 3 h sessions or six 4 h sessions, and the corresponding sessions were processed with two methods (#1 and #2). In the #2 method, the data were processed with SF-PPP, while the DD ambiguity was introduced into SF-PPP in method #2. The convergence time of each session for the two methods was analyzed. Note that the convergence time was defined as the elapsed time when the estimated coordinate errors in the North, East, and Up directions were all smaller than 10 cm.

## 4. Discussion

The dual-frequency observations of the stations TJCH and GTFD were used to compute the precise ionosphere delay and then interpolate the ionosphere delay for the single-frequency users of CMCJ, CMMZ, JSFJ, and NHSY. The interpolated ionosphere delay was analyzed, and the SF-PPP is realized based on the interpolated ionosphere delay. Then the introduction of the DD ambiguity into SF-PPP was implemented.

### 4.1. Analysis of the Interpolated Ionosphere Delay

**Figure 3:**
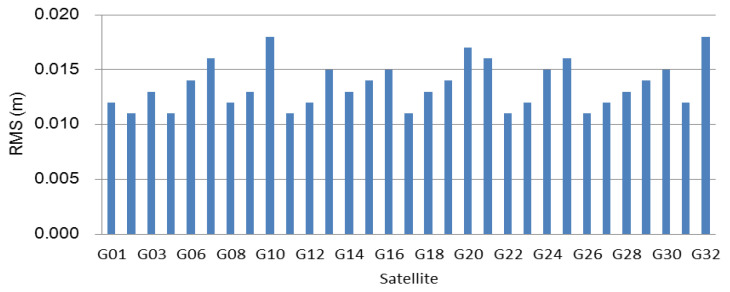
RMSs of the interpolated ionosphere delays for all tracked satellites at the reference epoch.

**Figure 4:**
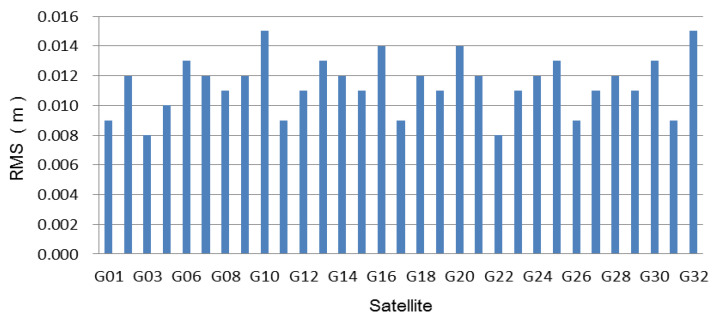
RMSs of the interpolated time-varying ionosphere delays for all tracked satellites.

The interpolated ionosphere delay was obtained using the proposed method in Section 2.2, and its performance was evaluated. Firstly, the reference epoch-based ionosphere delay was computed according to Equation (7). The RMSs of the difference between the interpolated ionosphere delay and the estimated values for all the satellites were computed and shown in Figure 3 and Figure 4. From Figure 3, it is observed that the accuracies of the interpolated ionosphere delay for all tracked satellites at the reference epoch are better than 2 cm, and the accuracies for most of the satellites reach 1.5 cm. Figure 4 shows that the time-varying interpolated ionosphere delay was better than the 1.6 cm, and the accuracies of most satellites were better than 1.2 cm. The interpolated ionosphere delay could be used to realize the SF-PPP.

### 4.2. Dual-Frequency Observations Enhanced SF-PPP

**Figure 5:**
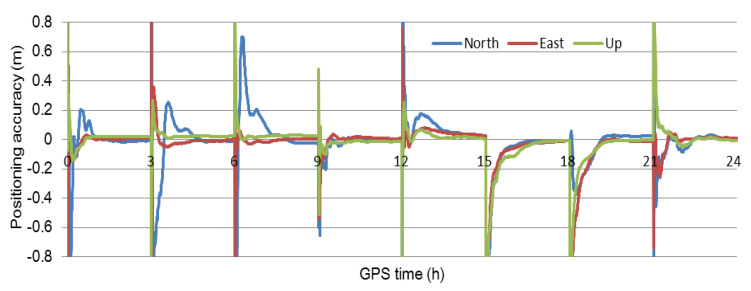
Positioning results of the user CMCJ for the eight 3-h sessions.

**Figure 6:**
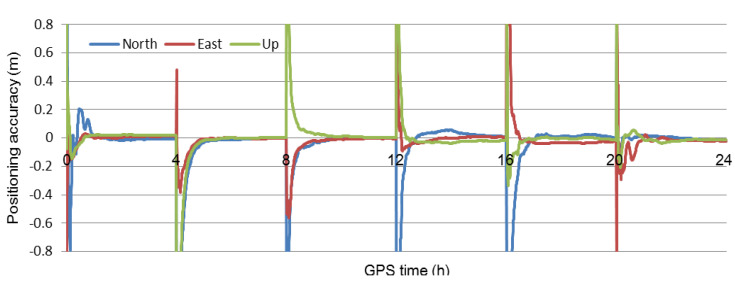
Positioning results of the user CMCJ for the six 4-h sessions.

According to the method for estimating and interpolating the precise ionosphere delay, the high-precision ionosphere delay at the user is obtained. Figure 3 and Figure 4 show that the accuracy of the interpolated ionosphere delay could reach the centimeter-level. When the precise ionosphere delay was obtained, the high-precision SF-PPP was realized. In order to obtain more results to analyze the performance of the proposed method, the 24 h data was split into eight 3 h sessions and six 4 h sessions. The dual-frequency observations enhanced SF-PPP results at the user CMCJ for eight sessions, and six sessions are shown in Figure 5 and Figure 6. The positioning results and convergence time for all users of CMCJ, CMMZ, JSFJ, and NHSY are shown in Table 2. As shown in Figure 5 and Figure 6, the SF PPP converged at a stable accuracy of centimeter-level after several minutes. These results also show that the convergence time of different observations was different. The positioning accuracy results in Table 2 were obtained by comparing the estimated results with that of the ground truth. The results in Table 2 show that the SF-PPP, in which the estimated and interpolated precise ionosphere delays were used, could reach centimeter-level positioning accuracy, although the average convergence time was 56.0 min. The average positioning accuracies in North, East, and Up directions reached 3.52, 3.53, and 4.11 cm, respectively.

When the DD algorithm was used, the relative positioning results were obtained. The relative positioning results could indeed be computed with the difference between the dual-frequency observations enhanced SF-PPP results of two stations. The relative positioning results of the users were analyzed to validate the advantage of the SF-PPP processing. The relative positioning results computed with two methods, the DD algorithm and the difference between the results of two stations estimated with SF-PPP, were compared. The relative positioning results of the two methods are shown in Table 3. The results showed that the relative positioning results of the DD algorithm were slightly better than that of the difference between the dual-frequency observations enhanced SF-PPP results. This could be explained by the effect of the float ambiguity resolutions. In the dual-frequency observations enhanced SF-PPP processing, the float ambiguity was estimated. This further validated the importance of ambiguity fixing for improving positioning accuracy.

### 4.3. Introduction of the DD Ambiguity into SF-PPP

**Figure 7:**
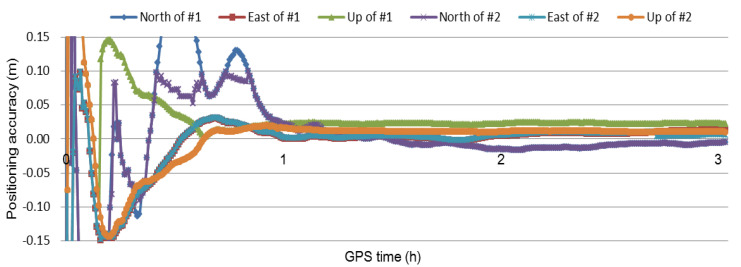
Positioning results of the user CMMZ for the first 3 h session.

It is well known that ambiguity fixing is beneficial for improving positioning accuracy. Generally, the FCB needs to be estimated and serviced in PPP ambiguity fixing. In Li et al. [6] and Odijk et al. [29], the DD ambiguity resolution [44,45] was introduced into PPP to realize PPP ambiguity fixing. This method avoids the complicated procedure of the FCB estimation. In SF-PPP fixing, the DD and PPP ambiguity resolutions are mutually employed. Here, during data processing, the realization of the SF-PPP ambiguity fixing was based on the DD ambiguity resolution. The quality of the SF-PPP float ambiguity result was very important in this processing. In fact, the application of the SF-PPP float ambiguity result was to cancel the effect of the FCB of the SF-PPP. The results of the user CMMZ for the methods of #1 and #2 are shown in Figure 7. Obviously, the result of the first 3 h session for method #2 was better than that of method #1 and the corresponding convergence time was shorter than that of method #1. The improved positioning accuracy and convergence time for all user stations and sessions are shown in Table 4. From Table 4, it can be seen that the mean improvement of the positioning accuracy in North, East, and Up directions were 0.63, 0.62, and 0.91 cm, respectively. The average convergence time for all user stations and sessions was 1.5 min. The improved accuracy and the convergence time validate that the introduction of the DD ambiguity resolutions into SF-PPP was meaningful for improving the performance of the SF-PPP. The accuracy improvement of the introduction of the DD ambiguity resolutions into SF-PPP could further improve the deformation monitoring ability of the SF-PPP.

To validate the meaning of the proposed method that introducing DD ambiguity into SF-PPP and demonstrate that this method can improve the positioning accuracy, the difference between the results of two SF-PPP stations were compared with the relative positioning results computed with the DD algorithm. The relative positioning results of the two methods are shown in Table 5. Table 5 shows that the relative positioning results of the DD algorithm and the difference between the results of using the SF-PPP, in which the ambiguity was fixed, were equal. This demonstrates that the method of the dual-frequency observations enhanced SF-PPP, in which the introduction of the DD ambiguity into SF-PPP was used, could improve the accuracy of the SF-PPP and the corresponding relative positioning results were equivalent to that of the DD algorithm.

## 5. Conclusions

The single-frequency GNSS receiver has been widely used. However, ionosphere delay error is one of the largest error sources of single-frequency positioning; it cannot be removed precisely and may result in range errors of several meters in the GNSS signal. It is common that the service of the ionosphere delay for the single-frequency user is realized by modeling the ionosphere delay estimated with code geometry-free combination formed with P1 and P2 observations from regional network stations. The accuracy of the serviced ionosphere delay is easily affected by the noise of the code observations. To obtain precise ionosphere delay, the phase observations of the dual-frequency observations are used to compute the time-varying ionosphere delay, and the code observations of the dual-frequency observations are used to compute the ionosphere delay at the reference epoch. The computed precise ionosphere delay can be used to interpolate the ionosphere delay at the user. However, the dual-frequency observations enhanced SF-PPP is still affected by the satellite FCB, and the corresponding ambiguity does not have integer characteristics. Thus, the ambiguity fixing for the dual-frequency observations enhanced SF-PPP was realized based on the introduction of the DD ambiguity into SF-PPP. The method for the introduction of the DD ambiguity into SF-PPP entails using the SF-PPP ambiguity at users to cancel the satellite FCB and to obtain the integer ambiguity. This integer ambiguity at SF-PPP user was the DD ambiguity formed with two user stations.

The collected data from six stations from Shanghai, China, was processed, and the corresponding results were analyzed to validate the proposed method. The computed and interpolated ionosphere delay reached the centimeter-level and was precise enough to realize SF-PPP. So, the static results of the dual-frequency observations enhanced SF-PPP reach the centimeter-level. The relative positioning results of the DD algorithm and the difference between the results of two stations estimated with dual-frequency observations enhanced SF-PPP showed that the results of the DD algorithm were slightly better than that of the dual-frequency observations enhanced SF-PPP. This could be attributed to the effect of the float ambiguity resolutions on the positioning accuracy. The data was processed with the presented method for the introduction of the DD ambiguity into SF-PPP, and the results indicate that this method improves the positioning accuracy and shortens the convergence time of the SF-PPP. The results can further verify the deformation monitoring ability of the SF-PPP. Although most of the new GNSS receivers receive multi-frequency and multi-system observations, some old single-frequency GPS receivers are still in use. The method proposed in this paper could be used for improving the positioning ability of these old receivers.

## Figures and Tables

**Table 1:** Adopted models and strategies for the SF-PPP.

	Items	Strategies
Measurements	SF code and phase observation;	L1;
	Adjustment;	Least-squares;
	Weighting.	Elevation-dependent function.
Corrections	DCB (P1-C1) and DCB (P1-P2);	Products provided by CODE
	Tide corrections;	Solid tide and Ocean tide correction [42];
	PCV;	Absolute IGS 08 correction mode
	Relativity.	Corrected.
Parameters	Station coordinates;	Estimated;
	Troposphere; Ionosphere delay;	Correction: Saastamoinen model [43]; Residual: Estimated in piece-wise mode; Correction: using the interpolated ionosphere delay of the dual-frequency observation;
	Receiver clock error;	Solved at each epoch as white noise;
	L1 ambiguity.	Float and fixed results.

**Table 2:** Positioning accuracy and convergence time.

Station		Positioning Accuracy (cm)			Convergence Time (min)
		North	East	Up	
CMMZ	3 h session	4.31	4.01	5.21	61.0
	4 h session	3.33	3.68	4.92	61.0
CMCJ	3 h session	3.76	3.82	4.01	55.0
	4 h session	3.56	3.41	3.99	56.0
NHSY	3 h session	3.61	3.52	3.85	53.0
	4 h session	3.26	3.12	3.67	53.5
JSFJ	3 h session	3.21	3.44	3.53	54.0
	4 h session	3.10	3.23	3.68	54.0
Mean		3.52	3.53	4.11	56.0

**Table 3:** Relative positioning accuracy of two methods.

Station		Observation Time	DD Algorithm (cm)			Difference between the SF-PPP Results (cm)		
			North	East	Up	North	East	Up
CMMZ	CMCJ	3 h session	1.31	1.56	1.86	1.49	1.96	2.06
		4 h session	1.02	1.13	1.65	1.32	1.43	1.95
CMMZ	NHSY	3 h session	0.98	1.06	1.12	1.18	1.27	1.33
		4 h session	0.66	0.87	0.96	0.79	0.93	1.13
CMMZ	JSFJ	3 h session	0.91	1.04	1.10	1.06	1.27	1.32
		4 h session	0.33	0.67	0.88	0.61	0.87	1.31
Mean			0.87	1.06	1.26	1.08	1.29	1.52

**Table 4:** Positioning accuracy and convergence time.

Station		Improved Positioning Accuracy (cm)			Improved Convergence Time (min)
		North	East	Up	
CMMZ	3 h session	0.68	0.53	0.95	1.0
	4 h session	0.78	0.64	1.11	1.0
CMCJ	3 h session	0.58	0.52	0.83	2.0
	4 h session	0.67	0.69	0.91	2.0
NHSY	3 h session	0.42	0.55	0.87	1.0
	4 h session	0.58	0.68	0.93	1.0
JSFJ	3 h session	0.61	0.63	0.77	2.0
	4 h session	0.74	0.71	0.92	2.0
Mean		0.63	0.62	0.91	1.5

**Table 5:** Relative positioning accuracy of two methods.

Station		Observation Time	DD Algorithm (cm)			Difference between the SF-PPP Results (cm)		
			North	East	Up	North	East	Up
CMMZ	CMCJ	3 h session	1.31	1.56	1.86	1.31	1.56	1.86
		4 h session	1.02	1.13	1.65	1.02	1.13	1.65
CMMZ	NHSY	3 h session	0.98	1.06	1.12	0.98	1.06	1.12
		4 h session	0.66	0.87	0.96	0.66	0.87	0.96
CMMZ	JSFJ	3 h session	0.91	1.04	1.10	0.91	1.04	1.10
		4 h session	0.33	0.67	0.88	0.33	0.67	0.88
Mean			0.87	1.06	1.26	0.87	1.06	1.26

## Data Availability

The data used to support the findings of this study are available from the corresponding author upon request.
